# Aging With Pride: Cultural Relevance of Motivational Interviewing for LGBTQ Adults With Dementia

**DOI:** 10.1093/geroni/igab046.428

**Published:** 2021-12-17

**Authors:** Ryan Petros, Karen Fredriksen Goldsen, Linda Teri

**Affiliations:** University of Washington, Seattle, Washington, United States

## Abstract

LGBTQ adults disproportionately experience dementia and are more likely to rely on informal social support to meet care needs in the community compared to cisgender, heterosexual peers. Culturally responsive interventions will accommodate the unique strengths and independence of this population and support self-determination as they navigate reduced capacity for self-care, increased need for support, and changes to autonomous, independent decision-making. Motivational Interviewing (MI) is a culturally responsive approach to treatment, amenable to integration with other evidence-based practices (EBP), and is especially relevant for the LGBTQ community for its compatibility with self-determination theory. This paper describes the unique contributions of MI that resulted in culturally relevant adaptations to a leading EBP for dementia and culminated in an efficacious intervention (Aging with Pride: IDEA) that is compatible with self-determination theory, designed for individuals and care providers in the LGBTQ community affected by dementia, and adapted for online delivery during the COVID pandemic.

